# Conquering Cancer Multi-Drug Resistance Using Curcumin and Cisplatin Prodrug-Encapsulated Mesoporous Silica Nanoparticles for Synergistic Chemo- and Photodynamic Therapies

**DOI:** 10.3390/nano12203693

**Published:** 2022-10-21

**Authors:** Prabhakar Busa, Ranjith Kumar Kankala, Jin-Pei Deng, Chen-Lun Liu, Chia-Hung Lee

**Affiliations:** 1 Department of Life Science, National Dong Hwa University, Hualien 97401, Taiwan; 2 College of Chemical Engineering, Huaqiao University, Xiamen 361021, China; 3 Department of Chemistry, Tamkang University, New Taipei City 251, Taiwan

**Keywords:** photodynamic therapy, p-glycoprotein, Cisplatin, mesoporous silica nanoparticles, reactive oxygen species

## Abstract

Recently, the development of anti-cancer approaches using different physical or chemical pathways has shifted from monotherapy to synergistic therapy, which can enhance therapeutic effects. As a result, enormous efforts have been devoted to developing various delivery systems encapsulated with dual agents for synergistic effects and to combat cancer cells acquired drug resistance. In this study, we show how to make Institute of Bioengineering and Nanotechnology (IBN)-1-based mesoporous silica nanoparticles (MSNs) for multifunctional drug delivery to overcome drug resistance cancer therapy. Initially, curcumin (Cur)-embedded IBN-1 nanocomposites (IBN-1-Cur) are synthesized in a simple one-pot co-condensation and then immobilized with the prodrug of Cisplatin (CP) on the carboxylate-modified surface (IBN-1-Cur-CP) to achieve photodynamic therapy (PDT) and chemotherapy in one platform, respectively, in the fight against multidrug resistance (MDR) of MES-SA/DX5 cancer cells. The Pluronic F127 triblock copolymer, as the structure-directing agent, in nanoparticles acts as a p-glycoprotein (p-gp) inhibitor. These designed hybrid nanocomposites with excellent structural properties are efficiently internalized by the endocytosis and successfully deliver Cur and CP molecules into the cytosol. Furthermore, the presence of Cur photosensitizer in the nanochannels of MSNs resulted in increased levels of cellular reactive oxygen species (ROS) under light irradiation. Thus, IBN-1-Cur-CP showed excellent anti-cancer therapy in the face of MES-SA/DX5 resistance cancer cells, owing to the synergistic effects of chemo- and photodynamic treatment.

## 1. Introduction

Cancer has emerged as one of the leading causes of death, accounting for millions of deaths each year worldwide [1]. The most commonly used treatment strategy, which employs traditional chemotherapeutics, is only partially effective due to low bioavailability and susceptibility to resistance acquired by cancer cells, resulting in poor therapeutic outcomes [2]. Furthermore, because of differences in pharmacokinetic (PK) and pharmacodynamics (PD) properties in the body, these agents frequently cause adverse effects [3]. Multidrug resistance (MDR) refers to a phenomenon in the cancer cells that can resist a variety of chemotherapeutic drugs through different molecular mechanisms [4]. Several studies on cancer biology have demonstrated that MDR is acquired through various approaches, including altered apoptosis pathways, enhanced deoxyribonucleic acid (DNA) damage repair, down-regulation of anti-apoptosis signaling pathways, alterations of drug entry at the cell membrane level via receptors, modifications of drug metabolism enzymes, and changes in cell membrane composition, etc. [5,6]. MDR is frequently developed through overexpression of adenosine triphosphate (ATP)-binding cassette (ABC) proteins such as MDR-associated protein-1 (MRP-1) and P-glycoprotein (P-gp) [7,8]. Many researchers have focused their efforts on overcoming resistance by targeting P-gp receptors, which effectively reduce the concentration of chemotherapy agents and various types of endo/exogenous toxins by relying on ATP levels [9]. Traditional approaches to MDR include the use of new ABC transporter inhibitors (small molecule or protein-based drugs) [10]. Thus, the development of ABC transporter inhibitors is a promising treatment for MDR by inhibiting the efflux receptors via antagonist action and impact on the functions of the efflux receptor [11]. To address these issues, researchers have focused their efforts on developing novel treatment modalities based on nanotechnology [12,13].

Nanoparticles as drug delivery systems have ushered in a new era in theranostics by employing various synthetic strategies to create multifunctional nanomaterials, combining various therapeutic and diagnostic approaches in a single formulation [14,15]. Because of their small size, targeted drug delivery, enhanced PK-PD profile, reduction in adverse effects, multiple drug delivery, and successful inhibition of efflux activity of P-gp receptors, these nanoconstructs have shown promising effects in overcoming the MDR of cancer cells [16]. Liposomes, polymeric cargos, solid lipid nanoparticles, dendrimers, carbon-based nanoparticles, layered double hydroxide nanoparticles, and MSNs have been used as a delivery vehicle to overcome MDR [17,18]. MSNs have received much attention because of their ease of synthesis, high surface area, high pore volume, and flexibility of surface functionalization, among other things [19,20,21,22]. Furthermore, using MSNs to deliver one or more drugs at a time improves the bioavailability of drugs with improved anti-cancer effects at low doses, reducing side effects [23,24,25]. Recently, some studies on the use of MSNs for treating MDR cancers using synergistic chemo-photodynamic effects have been published [26]. Previous research suggested that nanoparticles could avoid P-gp-mediated drug resistance by passing the efflux pump because these small-sized constructs could be easily internalized via endocytosis [27].

In recent years, some advanced therapeutic strategies, such as light-induced therapeutic strategies, have been used to address issues associated with simple drug delivery nanodevices and to improve therapeutic efficacy [28]. One of them is photodynamic therapy (PDT), which uses a specific light source to generate ROS by utilizing molecular oxygen to eradicate cancer cells [29]. This method allows for the selective destruction of cancer cells while leaving healthy surrounding tissues unaffected. Due to these facts, combining PDT with chemotherapy can significantly improve treatment efficacy against drug-resistant cancers [30]. Furthermore, combining PDT with chemotherapeutic drugs is highly effective in combating drug resistance at very low drug doses with minimal side effects [31]. ROS produced by PDT can permeate cell membranes, resulting in damage of DNA and protein, triggering apoptosis and necrosis of cancer cells [32]. The combination of PDT and chemotherapy can stimulate the synergistic effects and compensate for their shortcomings (photosensitizer agglomeration, drug efflux), thereby improving therapeutic outcomes [33]. Furthermore, by acting on several intracellular organelles such as mitochondria, endosomes, and DNA, combination therapies can potentiate ROS levels, alleviating drug resistance [34].

Accordingly, increasing ROS levels has become a powerful strategy for improving anti-cancer efficacy by combating drug resistance [12,13]. Several studies have been published, indicating that these nanoparticles are a highly effective strategy for combating drug resistance with minimal side effects [35]. For example, Tang and colleagues demonstrated the fabrication of magnetic species-functionalized MSNs to treat MDR breast cancer using doxorubicin (DOX) as a chemotherapeutic drug and Ce6 as a photosensitizer. This biocompatible formulation containing a drug combination demonstrated highly effective therapeutic effects at very low doses [36]. Zhang et al. recently developed an MSNs-based nanoformulation to deliver CP in combination with the photosensitizer chlorin e6 (Ce6) for the treatment of drug-resistant A549R lung cancer cell lines. The authors demonstrated that by enhancing ROS, this formulation successfully inhibited tumor growth and achieved very effective chemo-photodynamic therapy (CPDT) at a low dose [37]. Recently we published metal-substituted MSNs to improve the hydrophobicity and stability of drugs and to broaden the antibacterial therapeutic spectrum and address MDR by generating massive amounts of light-induced ROS [13,38].

In this study, we demonstrate the one-pot synthesis of Cur-embedded Institute of Bioengineering and Nanotechnology (IBN)-1-based MSNs using the triblock copolymer (F127) and fluorocarbon surfactants, motivated by these facts and the dreadful effects on MES-SA/DX5 cancer cells (MDR) cancer cells. The Cur species were first assembled in the micelle of the nonionic surfactant (F127). The silica source TEOS was then added, along with the particle size limiting of fluorocarbon surfactant (FC-4), to produce the IBN-1 structures. Furthermore, the diaqua prodrug of CP was immobilized on the surface of the carboxylate-modified MSNs. These nanocomposites were also systematically characterized using various techniques. Finally, the MES-SA/DX5 cancer cell (MDR) line was used to test the bio-efficacy of the designed nanoconjugates for chemo-photodynamic synergistic therapy. The rationale for choosing IBN-1, one type of mesoporous silica-based material, is that it is convenient for the encapsulation of dual drugs, specifically the hydrophobic curcumin drug. The triblock copolymer (F127) in the nanochannels would substantially hold the curcumin species. In addition, the particle size of IBN-1 is smaller than SBA-15 species and has larger pores compared to MCM-41 species [19,20,21]. Considering these attributes, IBN-1-based composites were preferred over other mesoporous silica-based materials in this study.

## 2. Materials and Methods

### 2.1. Materials

Cur (Purity: >75%), Pluronic F-127, [3-(2-aminoethylamino)propyl]trimethoxysilane (Purity: 97%), glutaric anhydride (GA, Purity: 95%), cis-diamine dichloro-platinum (II) (Purity: 99.9%), 1,3-diphenylisobenzofuran (DPBF, Purity: 97%), trypan blue solution (0.4%), o-phenylenediamine (OPDA, Purity: ≥98.0%), ninhydrin, and Thiazolyl Blue Tetrazolium Bromide (MTT, Purity: 98%) were purchased from Sigma Aldrich Co. Ltd. (St. Louis, MO, USA). Dimethyl sulfoxide (DMSO, Purity: ≥99.9%), dimethylformamide (DMF Purity: ≥99.9%), ethanol (EtOH), and toluene (Purity: 99%) were obtained from Acros Organics (Loughborough, UK). JC-1 assay kit was purchased from Invitrogen (Waltham, MA, USA).

### 2.2. Characterizations

To explore the chemical functionalities, the designed nanoparticles and their successive modifications of functional groups were subjected to Fourier-transform infrared spectroscopy (FT-IR) spectroscopy on the Bruker Alpha Spectrometer (Madison, WI, USA). The zeta potential values of analogous modified samples were measured by a Malvern Zetasizer (Zeta PALS, Malvern Panalytical, and Malvern, UK). Ultraviolet-visible (UV-Vis) absorbance values were recorded on a Genequant–1300 series spectrophotometer (GE Healthcare Biosciences, Pittsburgh, PA, USA). Flow cytometry analysis was performed on a C5 Flow Cytometer (BD Accuri™).

### 2.3. Cur-Embedded IBN-1 Synthesis

The IBN-1 nanoparticles embedded with Cur were synthesized using the previously described procedure with minor modifications [39]. 3.25 g of Pluronic F127, 1.2 g of Cur, and 4 g of FC-4 were dissolved in 200 mL of 20 mM HCl solution and stirred for 2 h at 30 °C, resulting in the encapsulation of Cur molecules in the Pluronic F127 micelle. In addition, 11 g of TEOS was added dropwise to the above solution and stirred at a constant temperature for 24 h. The total solution was hydrothermally treated by placing the resultant mixture in an autoclave at 100 °C for 24 h. The Cur-embedded IBN-1 solid product was centrifuged and thoroughly washed with an excess of dd-H_2_O to remove the surfactant FC-4. Finally, the IBN-1-cur product was lyophilized and stored at 4 °C for future experiments.

### 2.4. Diamine-Modified IBN-1-Cur Synthesis

To improve drug binding affinity on the surface of IBN-1 nanoparticles, we modified most outside surfaces of IBN-1-Cur with diamine silane at high temperatures with constant stirring [40]. 200 mg of IBN-1-Cur sample was first dispersed in 80 mL of dry-toluene and thoroughly mixed before continuing to stir at 80 °C. The homogeneous nanoparticle mixture was then added with 1 mL of diamine silane. The reaction was further stirred for 20 h under the same conditions. Furthermore, the solid product was centrifuged and washed with hexane twice before being dried under vacuum at room temperature. The product was denoted as IBN-1-Cur-2N.

### 2.5. Carboxylic Acid Group Modification

The acid modification was carried out following the previously reported procedure [41]. In 10 mL of dd-H_2_O, 100 mg of IBN-1-Cur-2N nanoparticles were suspended. The mixture was then mixed with 60 mg of GA at room temperature and stirred for 12 h at the optimal speed. Finally, the nanoparticle sample was collected and washed several times with water. The finished product was vacuum-dried and stored at 4 °C. The product was denoted as IBN-1-Cur-GA.

### 2.6. CP Coordination on IBN-1-Cur-GA

The highly reactive diaqua-CP was created by replacing the CP’s two chlorine atoms [42]. First, 50 mg of CP was placed in a centrifuge tube containing a solution (57 mg in 4 mL of dd-H_2_O). The mixture was shaken for 24 h at 37 °C in the dark in an arbitrary shaker. Furthermore, the silver chloride (AgCl) precipitate was centrifuged, the diaqua-modified CP-containing supernatant was carefully collected, and the pH of the sample was adjusted to 6.8. Finally, dd-H_2_O was used to increase the volume to 10 mL. The diaqua CP solutions were mixed with 50 mg of IBN-1-Cur-GA nanoparticles and shaken for 24 h at 37 °C in the dark. Centrifugation was used to collect the CP-coordinated nanoparticles, and the resulting solid was washed several times with dd-H_2_O to remove the unreacted drug molecules. Finally, the CP loading percentage was calculated by using UV-vis spectrophotometry to validate changes in absorbance values at 275 nm.

### 2.7. In Vitro Release Studies

The CP release from the IBN-1 nanocomposites was accomplished using the previously described procedure [43]. Initially, the CP calibration curve was established and mixed with OPDA solution with heating at 100 °C for 10 min, resulting in a light green color change. After cooling to room temperature, the solution was subjected to UV-vis spectrometry analysis at 705 nm to obtain the standard curve. The test sample solutions were then prepared by suspending 5 mg of CP-loaded IBN-1-Cur nanoparticles in 1 mL of phosphate-buffered saline (pH-7.4 and 5.0). Furthermore, aliquots of samples were separated at predetermined intervals, and the equivalent amount was replenished accordingly. Finally, the amount of CP released from the nanoconjugates was calculated by comparing it to the standard curve.

### 2.8. Ninhydrin Assay

In general, the traditional ninhydrin assay was used to determine both quantitative and qualitative primary amines [44]. The ninhydrin solution (in methanol) readily reacts via nucleophilic displacement reaction, which produced Ruhemann’s purple color upon heating with a maximum absorption peak at 570 nm. In a nutshell, the same amount of amine, acid, and CP-loaded IBN-1-Cur nanoparticles was mixed with 1 mL of ninhydrin solution. The tubes were then sonicated for 2 min before being heated at 90 °C for 20 min. Finally, the solid was removed by centrifugation, and the supernatant was analyzed with a UV-vis spectrophotometer.

### 2.9. Photostability Test

The stability of Cur-encapsulated nanoparticle samples after prolonged exposure to light illumination was investigated [45]. The IBN-1-Cur nanoformulation (5.0 mg) and equivalent molar concentration of Cur were weighed and dissolved in 1 mL of DMSO: H_2_O solution (1:1 *v*/*v*). The prepared samples were irradiated with a UV light source for 7 min, and the changes in absorbance values were recorded using UV-vis spectrometry at 1-min intervals. Notably, the experiment safety precautions were taken, such as placing the samples in the dark of 4 °C, to minimize the external thermal effect on the samples.

### 2.10. DPBF Assay for Detecting the Generation of Singlet Oxygen (^1^O_2_)

A steady-state method was used to detect the ^1^O_2_ species using the DPBF probe [46]. Indeed, the property of DPBF’s participation in the Diels-Alders reaction (1,4-cycloaddition) can be used as a sensitive probe for the detection of ^1^O_2_ species in the presence of a light source in vitro. Correlating the decreasing intensity of DPBF with the amount of ^1^O_2_ species allows us to calculate the amount of ^1^O_2_ species. In brief, 2 mg of various nanoparticles, IBN-1-Cur, and IBN-1-Cur-CP were dissolved in 10 mM of DPBF solution (EtOH:H_2_O, 1:1 *v*/*v*), and the mixture solution was illuminated with blue light (15 mW/cm^2^) for 5 min, and changes in absorbance values at 412 nm were recorded using a UV-vis spectrophotometer. Furthermore, the sample without the Cur-loaded IBN-1 nanoparticle and the DPBF solution alone served as a control experiment.

### 2.11. Cell Culture

The BioSource Collection and Research Center provided human uterine sarcoma drug resistance (MES-SA/DX5 cell line) cells (Hsinchu, Taiwan). In a humidified incubator (37 °C, 5% CO_2_), the cells were cultured in McCoy’s medium with 10% FBS.

### 2.12. In Vitro Cytotoxicity of IBN-1-Cur-CP against Drug-Resistant Tumor Cells

The drug-resistant MES-SA/DX5 cell line was used to test the in vitro cytotoxicity of IBN-1-Cur-CP using the MTT assay. The cells were seeded in a 96-well plate at a density of 1 × 10^4^ cells per well and incubated in a complete medium for one day. The cells were then treated with different concentrations of nanoparticles and incubated for 24 h. Similarly, the experiment was repeated by treating the cells with nanoparticles for 4 h, irradiated with 450 nm light (15 mW/cm^2^) for 5 min, and further incubated for another 20 h. Finally, the medium was added 10 μL of MTT reagent (5 mg/mL in PBS-7.4) and incubated for another 4 h. After removing the medium and washing the cells with PBS, the production of formazan crystals was dissolved by adding 100 μL DMSO. The optical density (OD) at 550 nm was measured using a microplate reader to determine cell viability. The following equation was used to calculate cell viability.

Cell viability (%) = (OD of treated group/OD of control group) × 100

### 2.13. Trypan Blue Cell Assay

We used a trypan blue exclusion assay, as described in the previous report [47], to determine the number of viable cells. In brief, the MES-SA/DX5 cells were cultured in a 6-well plate at a density of 1 × 10^5^ cells per well for one day. After that, the cells were treated with IBN-1-Cur-CP, exposed to light for 5 min, and incubated for 24 h. The trypan blue dye (10 μL) was added to the stain for 5 min and then washed with PBS twice. Images of cells were captured using a microscope to determine the distribution of dead and live cells.

### 2.14. DCFDA Test

The DCFDA fluorescent probe assay was used to detect the intracellular generation of ROS levels [48]. The MES-SA/DX5 cells were cultured for 24 h in a 6-well plate at a density of 1 × 10^5^ cells/well. In addition, IBN-1-Cur-CP (50 µg/mL) was added to the culture medium. After incubating for 4 h, 10 μM DCFDA was added to each well and incubated for another 30 min. The cells were carefully washed with PBS, further exposed to the light source for 5 min (PDT group), and then incubated for 12 h. Finally, the cells were observed using a fluorescence microscope (Ex/Em-488/525 nm), and the amount of DCF fluorescence produced by ROS was measured using flow cytometry.

### 2.15. JC-1 Test

The transmembrane mitochondrial membrane potential (MMP) was measured using the JC-1 assay as described previously [49]. MESA-SA/DX5 cells were harvested in 6-well plates at a density of 1 × 10^5^ cells per well, incubated overnight, and then treated with IBN-1-Cur-CP nanoconjugates for 4 h. The cells were carefully washed with PBS, and further exposed to the light source for 5 min (PDT group). After 12 h, 2 μmol/L of JC-1 fluorescent probe was added and incubated for another 30 min. A microscope and plate reader were used to estimate the fluorescence of each sample (JC-1 aggregate with red emission and. JC-1 monomers with green emission). MMP levels indicating mitochondrial health were quantified as fluorescence intensities ranging from red to green, which were then correlated with control cells. A fluorescent microscope was used to capture the images.

## 3. Results and Discussion

**Figure 1:**
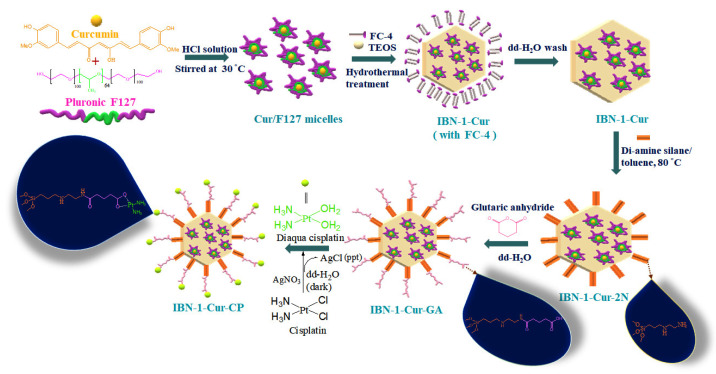
A schematic representation of the synthesis of IBN-1-Cur, subsequent surface modifications, and CP species loading.

Due to their unique properties, MSNs have garnered enormous interest from researchers for biomedical applications due to their ability to control the release of multiple drugs, ease of synthesis, control of size, and particle clearance through degradation under physiological conditions. In general, one of the most commonly used strategies for the synthesis of MSNs is a sol-gel process, which is known to impart good morphological properties in terms of size, shape, and surface area. In this paper, we show how to use acidic media to create MSNs with tunable morphology and size control for the delivery of hydrophobic drug cargo. Initially, a weakly acidic solution was used to dissolve the copolymer F127 and Cur, resulting in the formation of Cur-encapsulated micelles. The FC-4 surfactant was then combined with TEOS to form a mesoporous silica shell over the micelle (Cur@F127). In this context, the acidic medium can delay silica hydrolysis by stabilizing the silica shell over the Cur@F127 micelle and surrounding FC-4 surfactant molecules, limiting silica growth and yielding stable IBN-1-Cur nanoparticles. Furthermore, the surface was post-modified with amino functional groups by condensation at high temperatures of 3-(2- aminoethylamino) propyltrimethoxy silane) (diamine silane). Through a coupling reaction, the obtained IBN-1-Cur-2N surface was further reacted with GA. Finally, the surface of carboxylated groups was coordinated with diaqua CP (prodrug) for combined chemo- and photodynamic therapy to combat MES-SA/DX5 cancer cells’ drug resistance (Figure 1).

### 3.1. Synthesis and Characterization

The hydrodynamic size and polydispersity index (PDI) of the IBN-1-Cur and further their modified samples were measured by the dynamic light scattering (DLS) method, and the data are shown in Table 1.

**Figure 2:**
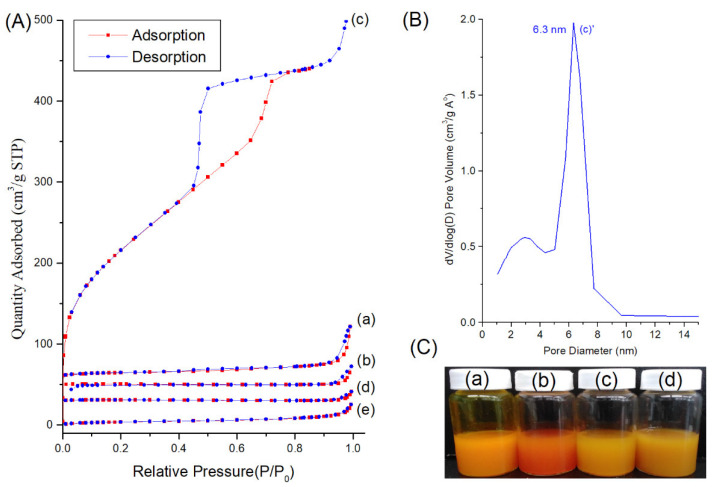
(**A**) Nitrogen adsorption–desorption isotherms of (a) IBN-1-Cur, (b) IBN-1-Cur-2N, (c) IBN-1-Cur-2N (calcination), (d) IBN-1-Cur-GA, and (e) IBN-1-Cur-CP samples. (**B**) Pore size distribution of the IBN-1-Cur-2N (calcined sample). (**C**) Photographic representation of aqueous solutions containing (a) IBN-1-Cur, (b) IBN-1-Cur-2N, (c) IBN-1-Cur-GA, and (d) IBN-1-Cur-CP samples.

The average diameter of IBN-1-Cur was 198.6 nm (PDI 0.107), diamine and glutaric anhydride-modified samples IBN-1-Cur-2N was 220.4 nm (PDI 0.128), IBN-1-Cur-GA was 239.5 nm (PDI 0.204), respectively. The particle size of these samples was increased by 21.8, and 40.9 nm, respectively, compared with IBN-1-Cur, which might be attributed due to the surface modifications of functional groups. The size of IBN-1-Cur-CP was 247.5 nm (PDI 0.124), a slightly increased size compared with glutaric anhydride functionalized formulation, attributing to the successful coordination of chemically modified CP prodrug with IBN-1-Cur-GA nanoparticles. The zeta potential of nanoparticles plays a critical role in the stability of nanoformulations in an aqueous medium. Because of the extensive surface silanol groups, the as-synthesized IBN-1-Cur formulation has a negatively charged zeta value of −14.12 mV. The IBN-1-Cur nanoparticles were then modified with diamine functional groups, yielding a positive zeta potential of +24.56 mV. The IBN-1-Cur-2N samples were then modified with a glutaric anhydride functional group (IBN-1-GA), which shifted the zeta value to a negative charge of −16.02 mV, indicating successful acid functional group conjugation. At neutral conditions, the negative charge could be attributed to the deprotonation of the surface with acid functional groups. Finally, IBN-1-Cur-CP samples have a positive zeta potential of +10.01 mV, indicating that the CP prodrug was immobilized on the surface. Because cancer cell membranes are negatively charged, the positively charged final formulations may enhance cellular affinity and uptake of the IBN-1 species. The nitrogen adsorption–desorption isotherms were used to measure the surface area, pore size, and pore volume of the IBN-1-Cur and their modified samples (Figure 2A). The results are summarized in Table 1. The surface area and pore volume of as-synthesized IBN-1-Cur, the surface-modified diamine silane, grafted GA, and further coordinated CP samples are very small. Notably, the main surface area and pore volume of mesoporous silica are contributed by the internal structure of the material itself. During the synthesis process, F127 and Cur are self-assembled to form a micelle structure. Thus, the as-synthesized product of silica frameworks can stabilize F127/Cur in the inner structures of IBN-1. Despite the continuous reaction and washing process, the Cur molecules are very stable inside the nanochannels and have no leaching phenomenon to maintain a high loading capacity. To further confirm the existence of mesoporous structure in the IBN-1-Cur sample, we used the diamine silane modified sample to calcine at 550 °C for 5 h for F127/Cur removal. We can find that the calcined samples showed a complete restoration of the IBN-1 pore structure with high surface area (830.6 m^2^/g), pore volume (0.824 cm^3^/g), and single pore size distribution. The pore size distribution was determined by the adsorption curve of the isotherm from Barrett–Joyner–Halenda (BJH) method. The curve of Figure 2B(c’) showed uniform pore size of a single distribution peak with a maximum value falling at 6.3 nm. The results showed that the one-pot reaction by the co-condensation approach could achieve high loading of Cur for therapeutic purposes. The aqueous distributions of all sample photographs were enclosed in Figure 2C, indicating that the samples were distributed well in the aqueous medium without aggregation. These snippets of evidence suggest that these formulations with well-controlled particle sizes could easily apply in nanoparticle-based drug delivery systems.

**Figure 3:**
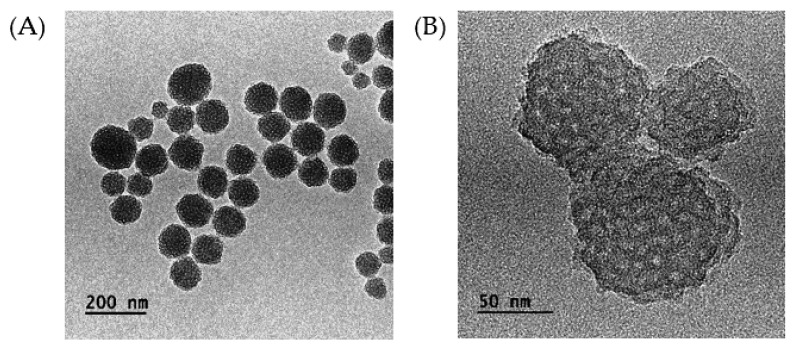
TEM images of (**A**) IBN-1-Cur-GA and (**B**) IBN-1-Cur-CP.

Transmission electron microscopy (TEM) images were also taken to confirm the morphology of the nanoparticles. TEM images of IBN-1-Cur-GA and IBN-1-Cur-CP samples are shown in Figure 3. The synthesized nanoformulations were found to possess a spherical shape and pore-dense matter, which could be attributed to the presence of F127@Cur micelles in the IBN pores. The nanoparticle size of the silica core was near 100–200 nm. Furthermore, IBN-1-Cur-GA and IBN-1-Cur-CP samples had a much rougher surface, indicating successful deposition of functional groups and CP prodrug on the surface.

**Figure 4:**
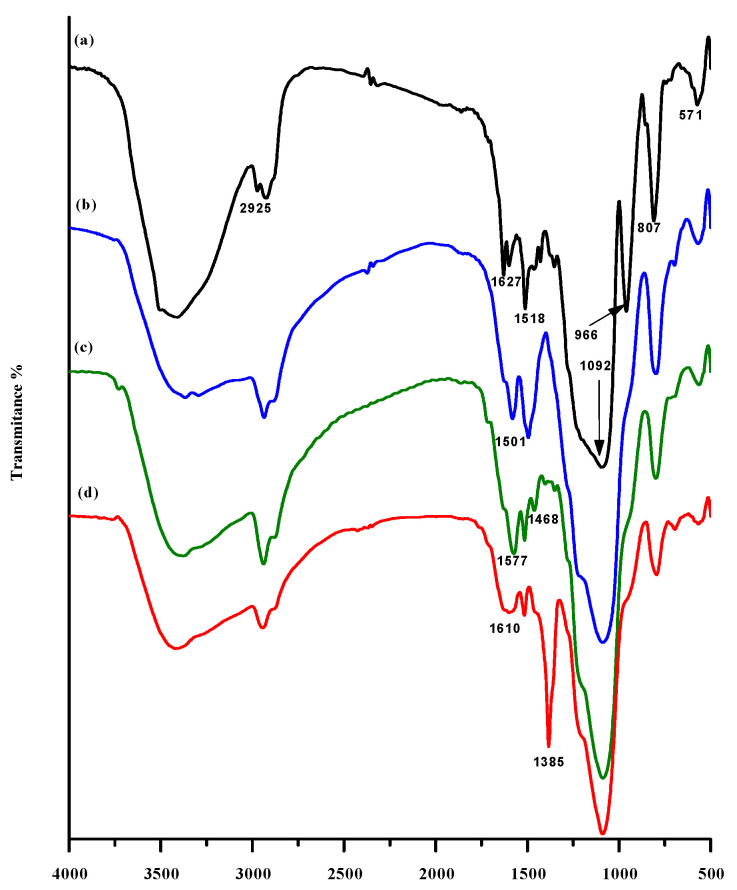
FT-IR spectra of; (a) as-synthesized IBN-1-Cur, (b) IBN-1-Cur-2N, (c) IBN-1-Cur-GA, and (d) IBN-1-Cur-CP.

Further, we used FT-IR spectroscopic investigations of Cur-embedded, amine-modified, acid-modified, and CP-loaded IBN-1 samples to demonstrate the successful immobilization of diverse organic functionalities (Figure 4).

The O-H stretching vibrations of adsorbed H_2_O molecules and hydroxyl groups of Cur species produced a broad spectrum at 3100–3500 cm^−1^ in the IBN-1-Cur sample. In its structure, the Cur molecule has three types of functional groups: two aromatic rings with O-methoxy phenolic groups attached to a seven-carbon linker with α, β-unsaturated, and β-diketone functional entities. Because Cur has a carbon backbone, the peak at 2925 cm^−1^ could be attributed to CH_2_ vibrations. This peak, however, may be overlapped with the carbon peaks in F127. Peaks at 1648 and 1545 cm^−1^ could be attributed to carbonyl functional groups, representing Cur’s tautomeric functional groups. To that end, the strong peaks at 1090 and 540 cm^−1^ could be attributed to silica peaks, such as Si-O-Si and Si-O-H peaks, which were observed in all samples, indicating the successful formation of a siliceous framework around the Cur@F127 polymer shell.

Furthermore, the IBN-1-Cur-2N sample exhibited a high-intensity peak at 1502 cm^−1^, which could be attributed to the N-H bending vibration of primary amines on the surface of nanoparticles, confirming successful diamine silane modification of the IBN-1 surface. Furthermore, GA samples showed a reduced peak at 1502 cm^−1^ and a new peak at 1578 cm^−1^, which could represent the C=O stretching vibrations of carboxyl groups (Figure 4c). The CP-loaded sample showed broad peaks at 3300 and 2400 cm^−1^, which could be attributed to OH molecules’ stretching vibrations. Furthermore, the N-H bending vibration of hydroxo-CP conjugated with the IBN-1-Cur-GA surface could represent a peak at 1610 cm^−1^ (Figure 4d).

**Figure 5:**
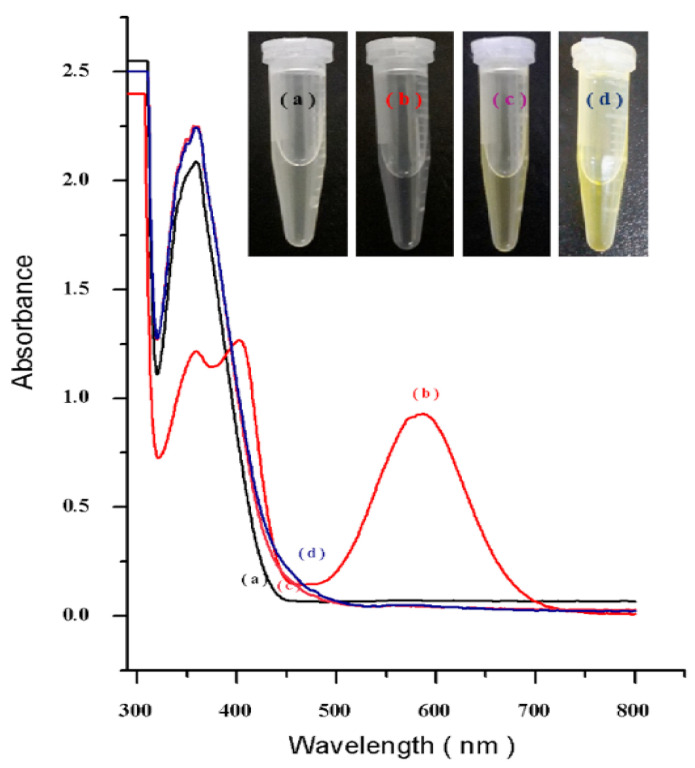
UV-vis ninhydrin absorption spectra of; (a) pure ninhydrin, (b) IBN-1-Cur-2N, (c) IBN-1-Cur-GA, and (d) IBN-1-Cur-CP samples. The photographs of the sample tubes are shown in the inset figure.

Figure 5 depicts the UV-vis spectrum of the ninhydrin test of various IBN-1-Cur functionalized surfaces, namely IBN-1-Cur-2N, IBN-1-Cur-GA, and IBN-1-Cur-CP samples. The amine-modified IBN-1-Cur-diamine sample exhibited Ruhemann’s purple color with peaks at 450 nm and 570 nm (Figure 5b), indicating the presence of primary anime functional groups that took part in the nucleophilic addition reaction with ninhydrin and produced the colorimetric product. In addition, the inset images confirmed Ruhemann’s purple coloring. In contrast, no characteristic absorption peak representing the ninhydrin complex was observed in the successively modified samples of GA and CP-immobilized nanoparticles, indicating that the amine functionalities were completely occupied

### 3.2. Photostability Studies

**Figure 6:**
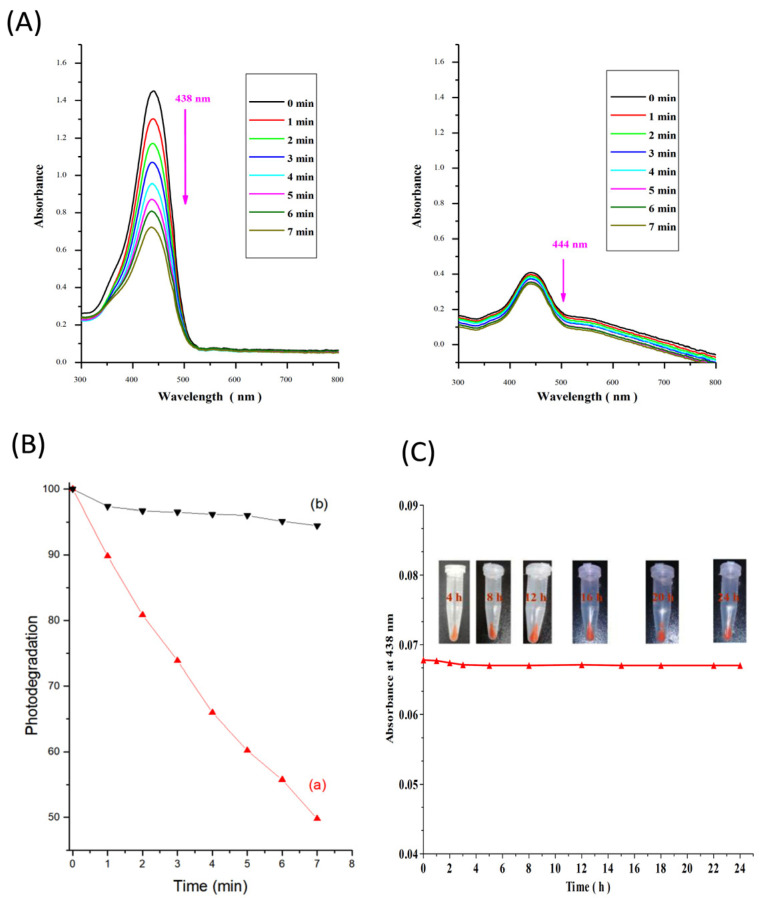
Photostability studies of Cur, (**A**) UV-vis spectrum analysis of absorption bands of free Cur and IBN-1-Cur-CP samples by UV light irradiation with time up to 7 min in DMSO: H_2_O (1:1 *v*/*v*) medium; (**B**) The samples of (a) free Cur (λ_max_ at 438 nm) and (b) IBN-1-Cur-CP (λ_max_ at 444 nm) irradiation under UV light up to 7 min, calculating the percentage photo-degradation rate; and (**C**) UV-vis absorbance values of Cur showing the leakage of Cur content from the IBN-1-Cur-CP formulation in cell culture medium for 24 h.

Despite the encapsulation of Cur species in nanoparticles, the stability of Cur in the presence of light is always a challenge. In this vein, it is clear from the literature that there are no obvious studies quantitatively explaining Cur’s Photostability [50]. In general, instability refers to the rapid degradation of Cur species in the presence of a light source, which may result in a loss of Cur’s bioefficacy. The aqueous and photo-stabilities of the IBN-1-Cur-CP formulation play critical roles in cancer therapy in evaluating the characteristic properties of PDT correlating to the efficacy of the formulation. First, the stability of IBN-1-Cur-CP in the presence of light was investigated. Figure 6A shows the UV-vis absorption spectra of free Cur and IBN-1-Cur-CP in DMSO: H_2_O (1:1 *v*/*v*) solutions irradiated with UV light at various time points. The intensity of the free Cur absorption peak (438 nm) decreased significantly as the light irradiation time increased from 0 to 7 min, but no apparent changes were observed in the IBN-1-Cur-CP samples (444 nm). The findings indicated that the Cur species embedded in the IBN-1 nanoparticles were highly stable in UV light. In addition, we tested the Cur stability in the IBN-1-Cur-CP nanoformulation by soaking it in a serum-free cell culture medium for 24 h and measuring the supernatant absorbance at 438 nm. The excellent aqueous stability was attributed to the fact that the release amount of Cur was meager. Further confirmation was obtained by photographing the insets of sample tubes at various time intervals. The experimental results revealed no significant differences, indicating Cur’s aqueous stability (Figure 6B,C).

### 3.3. CP Releasing Profile

**Figure 7:**
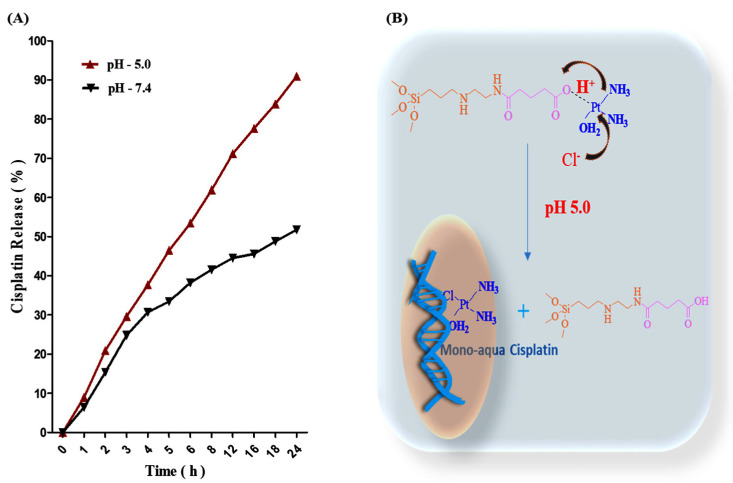
(**A**) The pH-dependent (pH 5.0 and 7.4) CP release for 24 h, (**B**) a diagram illustrating the mechanism of CP release in cancer cells, and highly active monoaqua CP binding to DNA.

Indeed, hydroxo CP molecules were conjugated onto IBN-1-Cur-GA samples via electron coordination in carboxyl groups and the d orbital of platinum. Through the endocytosis mechanism, the IBN-1-Cur-CP sample was trapped in endosomes. Because of the low pH gradient conditions, the interactions between carboxylated groups and CP would be weakened once the nanoparticles reached the endosome or late endosome. The nucleophile in the cytosol (Cl^−^ and H_2_O) may participate in the nucleophilic displacement reaction, producing highly reactive mono or diaqua (dichlorine) CP. The CP molecules could then bind to nuclear and mitochondrial DNA, inhibiting DNA synthesis, affecting transcriptional pathways and resulting in cell death. We used the OPDA assay to quantify the released amounts of CP in different pH values of PBS at 37 °C from the IBN-1-Cur-CP formulation (Figure 7A). The CP coordinated IBN-1-Cur-CP samples were dispersed in different pH of a PBS solution (pH-5.0 and 7.4) at 37 °C to mimic cellular endosome and cytosol environments in their releasing behavior. The resulting OPDA and released CP in DMF were heated at 100 °C for 10 min, resulting in a light green color and an absorption peak at 705 nm. The CP demonstrated an immediate release effect of around 29% in a pH-5.0 environment within 3 h, and the release reached 90% in 24 h. In the pH-7.4 environment, it showed around 51% in 24 h. The nucleophile attack in the acidic environment could explain the pH-dependent release of CP. These findings show that the specific release in an acidic environment would favor anti-cancer therapy (Figure 7B) [51].

### 3.4. In Vitro Singlet Oxygen Generation Assay

**Figure 8:**
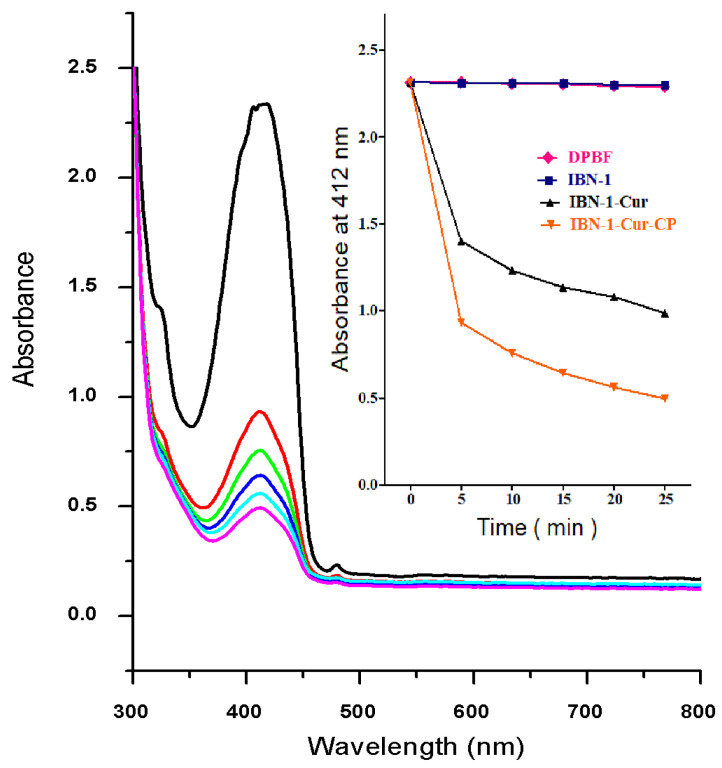
The photodegradation effect of DPBF through the generation of singlet oxygen species from IBN-1-Cur-CP exposed to light irradiation at different periods. The inset figure represents the time-dependent photobleaching of DPBF absorbance at 412 nm upon a light irradiation in the presence of IBN-1-Cur-CP at the time point of black-0, red-5, green-10, blue-15, cyan-20, and magenta-25 min.

Excessive intracellular ROS production has been linked to the destruction of DNA, cell membrane integrity, the abolition of lipid membranes, and the activation of apoptosis signaling pathways in cancer cells [52]. The DPBF assay was used to detect the levels of singlet oxygen species, a type of ROS, in the aqueous solution (EtOH: H_2_O, 1:1 *v*/*v*) to evaluate the potency of photodynamic features of IBN-1-Cur-CP. The UV-vis absorption intensities of DPBF were measured at 412 nm for samples of DPBF, IBN-1, IBN-1-Cur, and IBN-1-Cur-CP mixed with aqueous solution and exposed to light at various time points. Figure 8 shows that the absorption intensities of the IBN-1-Cur-CP sample decreased with increasing time up to 25 min, owing to the generation of ROS by Cur molecules under light irradiation. The absorption intensities of the IBN-1 and pure DPBF samples, on the other hand, were not changed significantly (Figure 8, inset).

### 3.5. Cell Viability Experiments In Vitro

**Figure 9:**
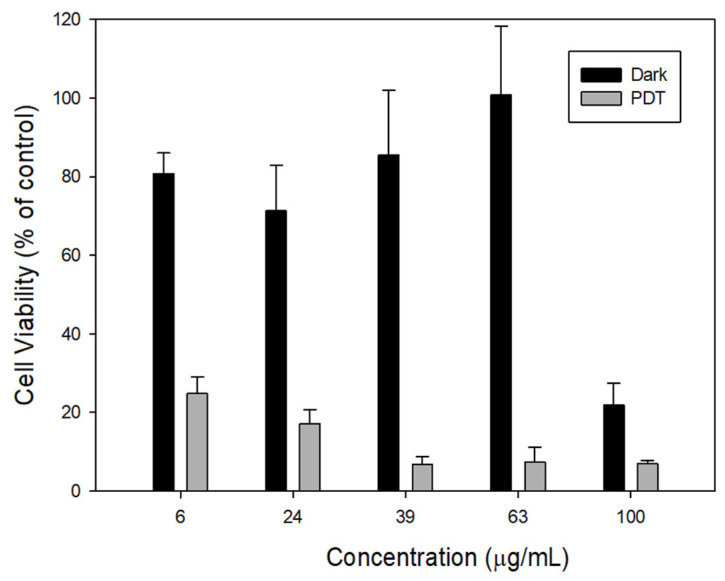
The cell viability of MES-SA/Dx5 cells treated with different concentrations of IBN-1-Cur-CP nanoformulation in the presence and absence of a light source.

Cur and CP are therapeutic agents that participate in the generation of ROS and induce cell death by altering the redox environments [53]. Furthermore, the F127 polymer inhibits efflux pumps, such as P-gp receptor efflux activity. It increases the concentration of therapeutic agents for the availability of producing the desired apoptosis activity to overcome MDR [54]. As a result, our formulation overcame MDR by successfully inhibiting the efflux activity of P-gp receptors and inducing cell death via enhanced ROS-mediated apoptosis via chemo- and photodynamic therapy [55]. To further demonstrate the efficacy of combination therapy, we used the MTT assay to assess the cytotoxicity of IBN-1-Cur-CP on drug-resistant MES-SA/DX5 cells. As shown in Figure 9, after 24 h of treatment with IBN-1-Cur-CP samples of varying concentrations, the designed nanoconjugates showed a significant reduction in cell viability in the presence of a light source versus without a light source.

### 3.6. Trypan Blue Assay

**Figure 10:**
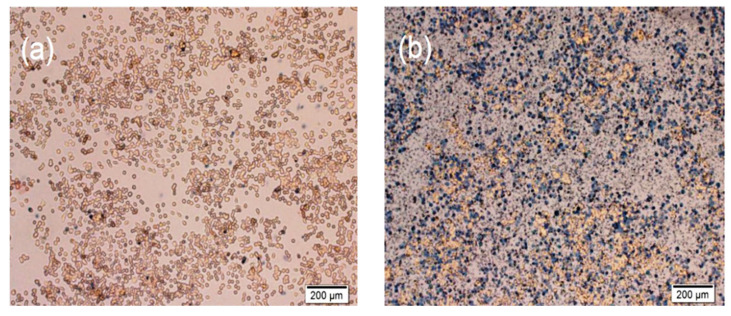
IBN-1-Cur-CP (50 µg/mL) treatment in MES-SA/Dx5 cancer cells in the (**a**) absence and (**b**) presence of light irradiation as measured by the trypan blue assay (scale bar 200 μm).

In addition, the trypan blue exclusion assay was used to assess the PDT effects of the nanoformulation. Trypan blue is a membrane-impermeable dye that can easily enter dead cells rather than living cells. Figure 10 shows that the cell membranes produced significant damage after treating IBN-1-Cur-CP (50 µg/mL) under light irradiation. Thus, many cells were stained by trypan blue dye, which was attributed to PDT effects from light-induced ROS generation [56].

### 3.7. In Vitro Cellular Uptake Studies

**Figure 11:**
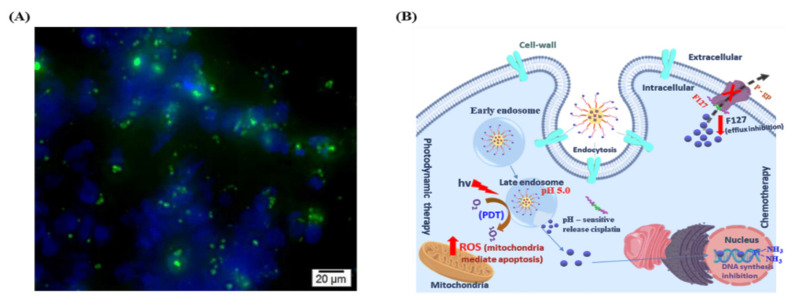
(**A**) IBN-1-Cur-CP nanoparticles (50 µg/mL) cellular uptake with DAPI nuclear staining (blue), scale bar 20 μm, and (**B**) schematic illustration of IBN-1-Cur-CP cellular uptake, pH-sensitive drug release, and involvement in chemo- and photodynamic therapy in MES-SA/DX5 cells.

Furthermore, the cellular internalization efficiency of IBN-1-Cur-CP was assessed using Cur’s self-fluorescence property (Figure 11A). In cases of endosomal/lysosomal pH sensitivity, nanoparticles enter cells via the endocytosis pathway and release therapeutic agents into the cytosol (Figure 11B). To demonstrate the delivery efficiency of the nanocomposites to MDR cancer cells, we treated the IBN-1-Cur-CP sample (50 µg/mL) to MES-SA/DX5 cells for 4 h, and the intracellular Cur fluorescence was measured using fluorescence microscopy. The nuclei were counterstained with DAPI, and the nanoparticles were identified using Cur species’ green fluorescence (Figure 11A). Microscopic studies showed large amounts of nanocomposites were uptake and accumulated in the cytosol of cells, and the green fluorescence was around the nucleus to demonstrate the successful internalization.

### 3.8. DCFDA Assay

**Figure 12:**
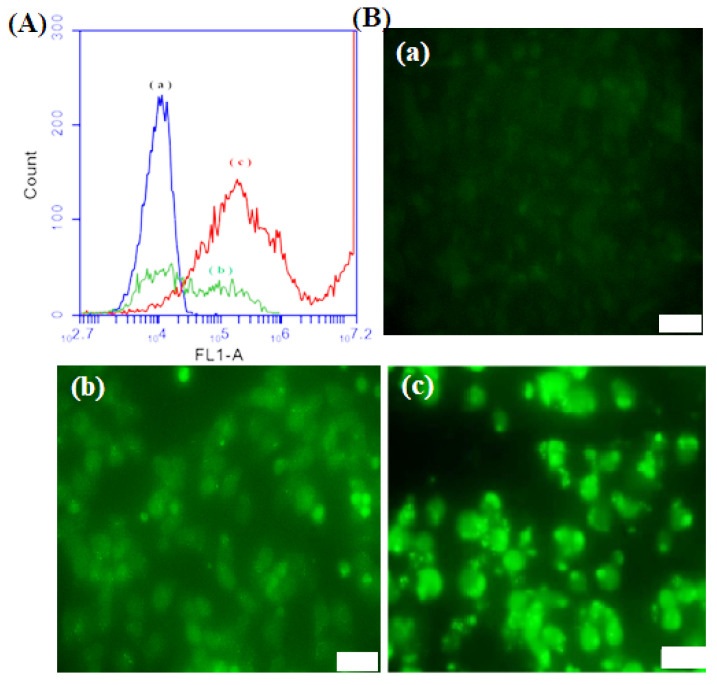
(**A**) Flow cytometry and (**B**) fluorescence microscopy of DCFDA assay to determine ROS in MES-SA/Dx5 cancer cells of (a) control; (b) IBN-1-Cur-CP, and (c) IBN-1-Cur-CP sample with light irradiation at the nanoparticle concentration of (50 μg/mL).

ROS levels in MES-SA/DX5 cells were determined using the DCFDA assay. In general, in the presence of ROS, the DCFDA probe is converted to the green fluorescent compound DCF (Figure 12). The cells were incubated with IBN-1-Cur-CP for 4 h and further exposed to the DCFDA and light source. The generated green fluorescence was observed under fluorescence microscopy.

Flow cytometry was used to quantify the intensity of cell fluorescence and determine ROS production (Figure 12A). Compared with the control group, the IBN-1-Cur-CP (Figure 12A(b)) treated cells showed higher fluorescent intensity with moderate ROS production. The further light irradiation of the IBN-1-Cur-CP sample could observe the highest fluorescence intensity with large amounts of ROS production (Figure 12A(c)). Microscopy images of cells treated with IBN-1-Cur-CP nanoparticles also revealed massive fluorescence, which was attributed to the production of excess ROS in (Figure 12B(a–c) MES-SA/DX5 cells. The findings support using IBN-1 as a delivery vehicle to achieve synergistic effects for chemo-photodynamic therapy against MDR cancer cells.

### 3.9. Assay for Mitochondrial Membrane Permeability (MMP)

**Figure 13:**
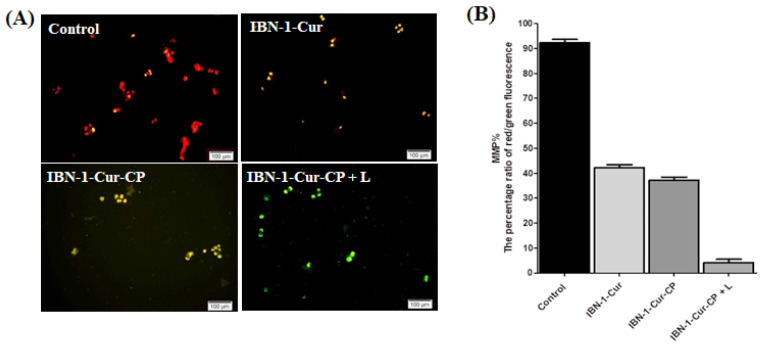
MMP assay on MES-SA/DX5 cells using the JC-1 staining method. (**A**) MMP depolarization was examined using a fluorescence microscope after cells were treated with IBN-1-Cur, IBN-1-Cur-CP (dark), and IBN-1-Cur-CP (light) (50 g/mL), a scale bar of 100 μm. (**B**) Quantitative analysis of the ratio of red/green fluorescent percentage intensity using a microplate reader.

The organelle mitochondria is a vital apoptosis target through lethal ROS production from therapeutic drugs [57]. The JC-1 fluorescent probe assay was used to estimate the potency of the IBN-1-Cur-CP sample on MMP (m) changes. At high MMP conditions, JC-1 shows an aggregated form and emits red fluorescence. However, the dysfunction of apoptotic cells shows depolarized mitochondria membrane, and JC-1 is monomeric and emits green fluorescence (Figure 13).

After treatment with the IBN-1-Cur sample (50 μg/mL), JC-1 emitted minor orange fluorescence and showed a slight decrease in the MMP. However, the IBN-1Cur-CP treated cells showed a further increase in fluorescent intensity, which could be due to the release of CP and the trigger of mitochondria ROS from the electron transfer chain. The transference of fluorescence from the red of the control group to the green of the IBN-1-Cur-CP sample (with light) indicated that nanocomposite treatments could result in a great decrease in MMP level. Via light irradiation, IBN-1-Cur-CP can further generate energy transfer of Cur to convert nontoxic triplet oxygen to cytotoxic singlet oxygen. Therefore, the green fluorescence demonstrated the trigger collapse and depolarization in the MMP (Figure 13A). Notably, combining chemo- and photodynamic therapy could enhance the nanoparticles to change in membrane potential of mitochondria and activate the intrinsic apoptotic pathway. To compare the effects of IBN-1-Cur and IBN-1-Cur-CP nanoparticles on MMP changes, the ratio of red/green fluorescence was determined using a microplate reader. As shown in Figure 13B, the control ratio of red/green fluorescence percentage was 92% after treatment with IBN-Cur (42%), IBN-1-Cur-CP (38%), and IBN-1-Cur-CP+L (4%).

## 4. Conclusions

In conclusion, we demonstrated the one-pot synthesis of Cur-embedded IBN-1 nanoconjugates, the successful immobilization of CP prodrug, and the conquering of MDR in MES-SA/DX5 cancer cells. The engineered IBN-1-Cur-CP nanoconjugates were systematically investigated with various physiochemical characterization methods, confirming their morphological, textural, and photostability attributes. Furthermore, the cellular experiments revealed an excellent internalization effect through endocytosis and increased ROS under the light source. The ROS enrichment strategy demonstrated significant anti-cancer activity against drug-resistant MES-SA/DX5 cells. Together, our findings indicated that these designed nanocomposites showed great potential against MDR cell line using a combination of chemotherapy and photodynamic therapy.

## Figures and Tables

**Table 1:** IBN-1 and their modified samples display surface area, pore volume, particle size, polydispersity index, and zeta potential.

Sample	Surface Area ^a^ (m^2^ g^−1^)	Pore Volume ^b^ (cm^3^ g^−1^)	Particle Size (nm)	PDI	Zeta Potential (mV)
IBN-1-Cur	17.2	0.095	198.6	0.107	−14.12
IBN-1-Cur-2N	0.8	0.035	220.4	0.128	+24.56
IBN-1-Cur-2N-C ^c^	830.6	0.824	N.D. ^d^	N.D. ^d^	N.D. ^d^
IBN-1-Cur-GA	2.6	0.017	239.5	0.204	−16.02
IBN-1-Cur-CP	13.9	0.039	247.5	0.124	+10.01

Note: ^a^ Determined by the BET method, ^b^ Determined by the t-plot method, ^c^ Calcined sample of IBN-1-Cur-2N, and ^d^ N.D. = Not determined.

## Data Availability

Not applicable.
